# The role of microbiota in the pathogenesis of schizophrenia and major depressive disorder and the possibility of targeting microbiota as a treatment option

**DOI:** 10.18632/oncotarget.21284

**Published:** 2017-09-27

**Authors:** Fengli Lv, Suling Chen, Lina Wang, Ronghuan Jiang, Hongjun Tian, Jie Li, Yudong Yao, Chuanjun Zhuo

**Affiliations:** ^1^ The department of rehabilition, The Second Affiliated Hosptial of Tianjin Medical University, Tianjin, China; ^2^ Department of Psychiatry, Wenzhou Seventh People’s Hospital, Wenzhou, Zhejiang, China; ^3^ Department of Psychiatry, Tianjin Anding Hospital, Tianjin Mental Health Center, Tianjin, China; ^4^ Department of Psychological Medicine, Chinese People’s Liberation Army, General Hospital, Chinese People’s Liberation Army Medical School, Beijing, China; ^5^ Department of Pharmacology and Physiology, SUNY Downstate Medical Center, Brooklyn, NY, USA

**Keywords:** microbiota, brain-gut axis, psychiatric disorders, schizophrenia, depression

## Abstract

The importance of interactions between the brain and the gastrointestinal tract has been increasingly recognized in recent years. It has been proposed that dysregulation and abnormalities in the brain-gut axis contribute to the etiology of a variety of central nervous system disorders. Particularly, dysbiosis, or impaired microbiota, has been implicated in multiple neurological and psychological disorders. The present paper reviews current evidence and theories concerning the possible mechanisms by which microbiota dysfunction contributes to the pathogenesis of schizophrenia and major depressive disorder. Clinical trials that investigated the possibility of treating both illnesses by correcting and rebalancing microbiota with probiotics are also reviewed. Overall, despite the accumulated knowledge in this field, more studies are warranted and required to further our understanding of the brain-gut axis and the possibility of targeting microbiota as a treatment option for schizophrenia and major depressive disorder.

## INTRODUCTION

The brain-gut axis conducts signaling between the brain and the gastrointestinal tract, which plays a fundamental role in normal physiological processes [1]. The human gastrointestinal system hosts approximately 1,800 different phyla and 40,000 bacterial species [2], collectively known as microbiota. Despite this substantial diversity, genetic analyses of fecal samples from a variety of individuals have indicated that intestinal bacteria form three distinct clusters, or enterotypes, suggesting that only a limited number of well-balanced and defined microbial community compositions exist across different individuals [3]. As gut microbiota plays an important role in host metabolism and intestinal inflammation responses, dysregulation of microbiota, or dysbiosis, can play a key role in the pathogenesis of inflammatory diseases in the host [4–6] and can serve as an alternative target for the treatment of inflammatory bowel diseases [7].

In recent years, interactions between the brain and gut microbiota have been explored, as microbiota has been found to be an important player in the pathogenesis of various neurological diseases [8] including multiple sclerosis [9, 10], Alzheimer’s disease [11], and Parkinson’s disease [12]. Indeed, microbiota can impact the structure of the brain. Myelination in the prefrontal cortex was found to be upregulated in a line of germ free mice [13]. More recently, Labus et al described a correlation between gut microbial composition and regional brain volume in patients with irritable bowel syndrome [14]. Furthermore, bacteria-derived metabolites could affect the CNS expression of brain-derived neurotrophic factor (BDNF) and other proteins that are important in cognition, which in turn affects host behavior [15]. The brain modulates gut functions including motility, acid secretion, the production of bicarbonates as well as mucus, and the immune response via the autonomic nervous system (ANS), thereby inducing responses to stress in the gastrointestinal system [16]. Particularly, the ANS-mediated modulation of mucus secretion could alter the environments that the microbiota inhabits and profoundly affect its composition and structure [17]. Therefore, the brain and the gut may form a feedback loop mediated by modulation of the microbiota. Mounting evidence suggests that gut microbiota plays a key role in many neuropsychiatric disorders [18, 19]. This review discusses the possible role of gut microbiota in the pathogenesis of schizophrenia and major depressive disorder (MDD), and further summarizes clinical data where the microbiota was targeted as a potential treatment method.

### Pathogenesis of schizophrenia and the involvement of gut microbiota

Schizophrenia is a serious psychiatric disorder, characterized by psychosis, in which the involvement of the gut-brain axis has long been recognized [20, 21]. Analyses of the microbiota in oral samples from schizophrenia patients found that the microbiota of these patients were comprised of significantly more *Lactobacillus* than normal controls [22, 23]. Further analyses indicated that increases in *Lactobacillus* group bacteria were significantly correlated with the severity of different symptom domains in schizophrenia patients [24]. Based on these studies, a relationship very likely exists between the microbial composition and schizophrenia.

The pathogenesis of schizophrenia is not fully understood; however, a decrease in BDNF expression and the resultant hypoactivity in N-methyl-D-aspartate (NMDA) receptor [25] have been implicated in the pathology of Schizophrenia. In a recent study Li et al first described a decrease in NMDA receptor expression in an animal model, and then by treating the animals with LY39, an agonist of mGluR, corrected the disrupted NMDA receptor expression. Furthermore, they observed that learning deficits and cognitive flexibility improved in treated animals, and thus found a correlation between the expression level of BDNF and NMDA and the symptoms of schizophrenia [26]. In a germ free mice line, decreased expression levels of BDNF and NMDA receptors were found in the cortex and hippocampus [27]. In schizophrenia patients, a decreased level of plasma BDNF has been noted, although findings from different studies have been inconsistent [28, 29]. Overall, the possibility that the microbiota affects BDNF expression and contributes to the development of schizophrenia warrants further investigation.

Converging lines of evidence indicate that the immune system could play an important role in the etiology of schizophrenia. Features of an autoimmune process, as well as diffuse non-specific immune system overactivation and activation of different types of T-helper cells, have been found in subgroups of schizophrenia patients [30]. A link between schizophrenia and an inflammatory-immune response has been further corroborated by the findings that antipsychotics produce anti-inflammatory effects in schizophrenia patients [31] and that non-steroid anti-inflammatory drugs could reduce the severity of schizophrenia type symptoms, especially in patients with a relatively more strongly altered immune response [32–34]. Troll-like receptors (TLR) mediate the innate immune response. TLR4 responds to lipopolysaccharides (LPSs), an important component of the outer membrane of Gram-negative bacteria, and triggers an inflammatory response [35]. Because TLRs are involved in the maintenance of intestinal epithelial homeostasis and protection from injuries as a result of the immune response to commensal bacteria, TLRs recognize the gut microbiota in healthy subjects [36, 37]. However, abnormal interactions between TLRs and gut microbiota could lead to chronic inflammation [37]. Thus, changes in the composition of microbiota as a feature of schizophrenia might induce an immune response mediated by TLRs and contribute to symptoms of the disorder.

Dysregulation in the metabolic pathway of tryptophan has been implicated in the pathogenesis of schizophrenia. Kynurenic acid is a tryptophan metabolite and an NMDA receptor antagonist [38]. An increased level of kynurenic acid in the central nervous system has been found in schizophrenia patients [39, 40]; however, a deficit of peripheral kynurenic acid has been proposed to be associated with a relapse [41]. Furthermore, an increase in the level of anthranilic acid, a downstream metabolite of kynurenic acid, has been found in schizophrenia patients, particularly a subgroup of patients with autoimmune presentation [42]. Interestingly, one study reported that the immune response to gluten, as assessed by measurement of serum antigliatin IgG level, was positively correlated with the kynurenine level and the kynurenine/tryptophan ratio [43]. Gut microbiota can substantially influence the level of plasma tryptophan [44–46], and thereby affect tryptophan metabolism. Although the exact role of the kynurenic pathway of tryptophan metabolism in the pathogenesis of schizophrenia has not been elucidated, some studies report that the upregulation of the pathway is associated with schizophrenia [47, 48]. Taken together, there is evidence pointing to the possibility that increased kynurenine level in the CNS is associated with Schizophrenia, and that this increase could lead to downregulation of NMDA activity or promotion of an immune response.

The cause of dysbiosis has also been investigated. As it is widely believed that an infant’s first exposure to bacteria is to the mother’s microbiota during vaginal delivery, it has been speculated that dysbiosis could result from a lack of exposure that occurs in a cesarean section birth. However, further analyses did not find that birth by cesarean section was correlated with schizophrenia [49, 50]. Nevertheless, the development of gut microbiota is shaped by early-life events, including mode of delivery, type of feeding, the application of antibiotics to the mother during delivery, and the gender of the infant [51–53]; therefore, the link between schizophrenia and dysbiosis requires further examination.

### Pathogenesis of MDD and gut microbiota

Mounting evidence supports the existence of a correlation between gut microbiota and the development of MDD [54, 55]. A study of fecal samples from MDD patients documented the occurrence of over- and under- presentation of the orders of *Bacteroidales* and *Lachnospiraceae*, respectively*.* The study further described a significant correlation that existed between depression and one clade in each of the genera *Oscillibacter* and *Alistipes* [56]. Subsequent analyses found that the bacteria that are associated with MDD were also associated with an increased level of isovaleric acid [56, 57]. Previous studies have shown that this colon-derived, short-chain fatty acid acetate can cross the blood-brain barrier and interact directly with the hypothalamus and the central homeostasis mechanism [58]. Moreover, valeric acid can act as an inverse agonist of the adenosine A_1_ receptor and influence the release of neurotransmitters in the brain [59]. Taken together, these studies indicate that changes in the gut microbiota may lead to an altered level of valeric acid, which in turn can affect the brain and contribute to the development of MDD.

Another recent study confirmed that microbial composition in MDD patients differs from that of non-depressed individuals. Analyses of fecal samples from MDD patients found a weak negative correlation between the relative abundance of *Faecalibacterium* and the severity of the depressive symptoms, and that the MDD patients had increased levels of Enterobacteriaceae and *Alistipes* but reduced levels of *Faecalibacterium* [60]. While the results of these studies are not completely consistent, for example, the variations in the reported gut bacterial species that are associated with MDD, they all underscore the connection between microbiota and MDD.

The level of tryptophan, a precursor of serotonin, is influenced by gut microbiota [44–46]. Specifically, in an animal study, treatment with *Bifidobacteria infantis* resulted in a significant change in serotonin metabolism in the brain [44]. The role of serotonin and its receptors in MDD has long been established [61, 62], and gut microbiota probably contribute to the development of MDD by affecting the tryptophan level.

Additionally, intestinal dysbiosis and a resultant “leaky gut” condition can lead to an increased immune response, and contribute to the etiology of MDD [63]. Given the role gut microbiota plays in the gut inflammatory response, it is highly likely that gut microbiota contribute to the pathogenesis of MDD by influencing the immune system and the inflammation response.

Further investigation of the correlation between MDD and the composition of microbiota is required to gain a better understanding of the mechanisms by which microbiota contribute to mood disorders.

**Figure 1:**
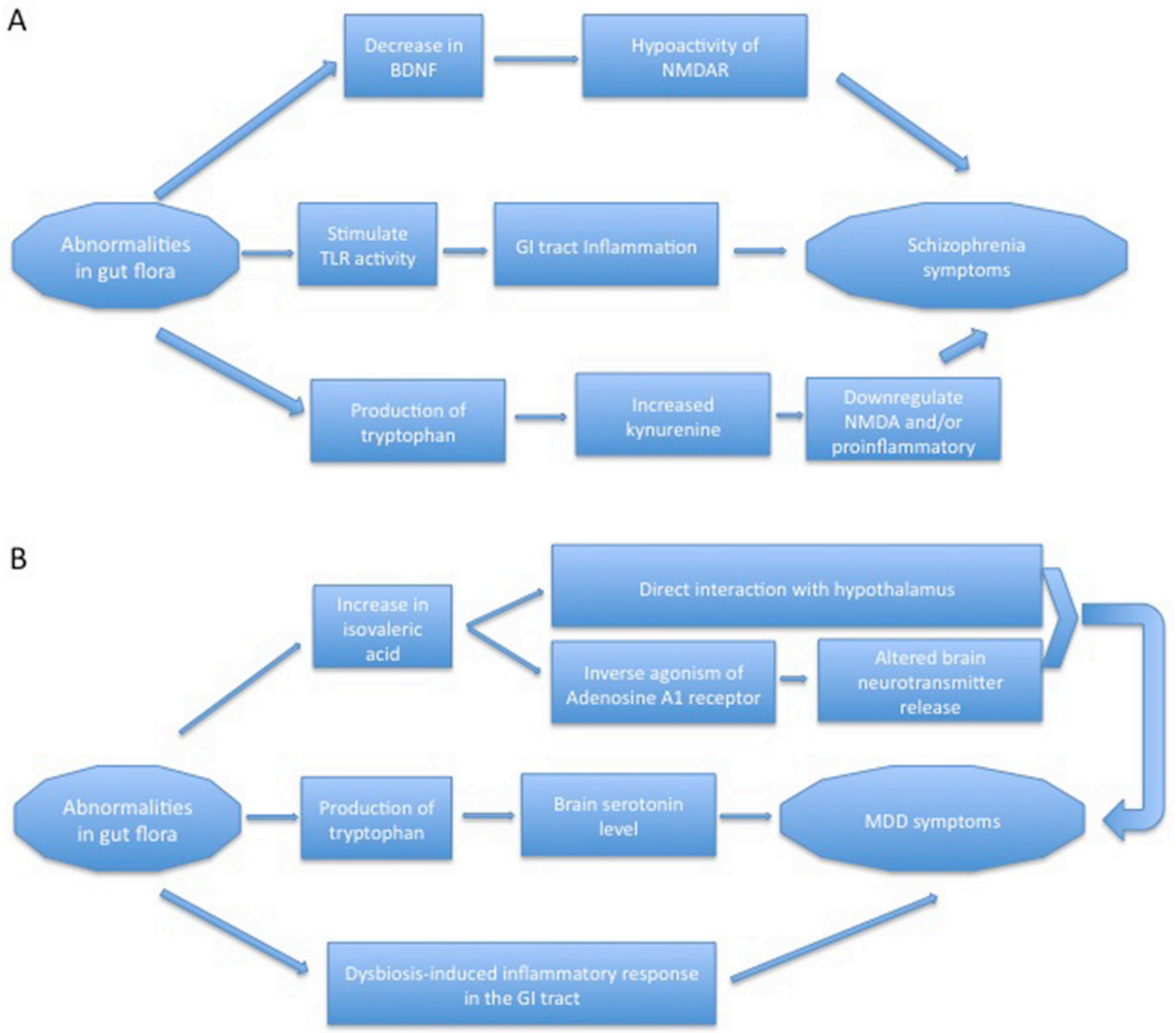
Possible mechanisms of gut microbiota in the pathogenesis of schizophrenia and MDD (**A**) Gut flora possibly contributes to the pathogenesis of schizophrenia through modulation of BDNF, the immune response in the gut, and the kynurenine pathway of tryptophan metabolism. (**B**) Gut flora possibly contributes to the pathogenesis of MDD through altering the levels of valeric acid and tryptophan, and the gut immune response.

Overall, gut flora may contribute to the pathogenesis of schizophrenia and MDD through the production of key molecules and/or promotion of intestinal immune response (Figure 1).

### Targeting microbiota in major psychiatric disorders

As we gain a clearer understanding of the relationship between dysbiosis and the development of schizophrenia and MDD, initial steps are being taken to explore the possibility of targeting microbiota as treatment options for both [64]. This review focuses on the use of probiotics in clinical trials that assessed the clinical benefits of probiotics in the treatment of schizophrenia and MDD. Relevant clinical trials are summarized in Table 1.

**Table 1:** Clinical trials with probiotics in the treatment of schizophrenia and MDD

Study	Population	Design	Intervention	Outcome
Schizophrenia				
Dickerson FB, et al., 2014 [63] ^*^	Schizophrenia patients	Randomized, placebo-controlled	Adjunctive probiotics vs placebo	No improvement in Schizophrenia symptoms, but improved GI functions
Tomaskik J, et al., 2015 [64] ^*^	Schizophrenia patients	Biomarker analysis	Adjunctive probiotics vs placebo	GI function improvement might be mediated by modulation of the immune response
Severance EG, et al., 2017 [65] ^*^	Schizophrenia patients	Longitudinal study	Adjunctive probiotic vs placebo	GI function improvement associated with a decrease in *C. albicans* IgG; a trend of improved positive symptoms
MDD				
Benton D, et al., 2007 [66]	Healthy volunteer	Double-blind, placebo-controlled	Probiotics vs placebo	Mood improvement in participants in the probiotic group who had poor mood initially
Messaoudi M, et al., 2011 [67]	Healthy volunteer	double-blind, placebo-controlled, randomized parallel group study	Probiotics	Improved psychological distress
Chung Y-C, et al., 2014 [68]	Elderly volunteer	double-blind, randomized	Fermented milk	Did not improve depression
Akkasheh G, et al., 2016 [69]	MDD patients	Double-blind, placebo-controlled	Supplement probiotics vs placebo	Significantly improved MDD symptoms
Bambling M, et al., 2017 [70]	SSRI-resistant MDD patients	Cohort study	Combined supplement of probiotics and magnesium orotate, co-administered with an SSRI	Significantly improved MDD symptoms, and relapse when patients ceased to take probiotics while on SSRI
Romjin AR, et al., 2017 [71]	MDD patients	Double-blind, randomized placebo-controlled	Probiotics as a primary treatment	No effect was noted in any of the psychological and functional assessments

MDD: major depressive disorder; SSRI: selective serotonin reuptake inhibitor

^*^The trials are connected and analyzed the same intention-to-treat population

An initial randomized, placebo-controlled clinical study investigated the effects of supplemental probiotics on the symptoms of schizophrenia and gastrointestinal function [65]. Recruited schizophrenia patients were given colony-forming adjunctive probiotics (*Lactobacillus rhamnosus* strain CG and *Bifidobacterium animalis* subsp. *lactis* Bb12) or placebos for 14 weeks. The study found that while offering no improvement in schizophrenia symptoms, administration of adjunctive probiotics was associated with improved gastrointestinal function. Biomarker analysis of the study participants found that the beneficial effects of probiotics are possibly mediated by modulation of the intestinal immune response [66]. A subsequent longitudinal study of the same population showed that the level of *C. albicans* IgG in male patients was significantly lowered, which was associated with improvements in the gastrointestinal complaints of male patients [67]. That study also reported improvement trends in the positive symptoms of schizophrenia [67]. Although the results from the clinical trials were promising, given the paucity of clinical data, the true efficacy and clinical benefits of probiotics in the treatment of schizophrenia patients remain to be validated by future clinical studies.

Probiotics have been shown to improve mood in the healthy population. A double-blind, placebo-controlled trial investigated the effects of probiotics on mood [68]. Healthy volunteers were given daily milk drinks containing *Lactobacillus casei* or a placebo for three weeks. At the end of the study period, the subgroup of participants receiving probiotics, whose mood was initially poor, showed improvements. In another study, the administration of probiotics (*L. helveticus* R0052 and *B. longum* R0175) reduced anxiety-like behavior in a murine model and improved psychological distress in healthy volunteers [69]. However, Chuang et al. reported that in healthy elderly Korean volunteers, the consumption of *Lactobacillus helveticus*-fermented milk was not associated with improved depression outcomes, as assessed with the geriatric depression scale -short form [70].

Studies that investigated the clinical benefits of probiotics in MDD have also yielded conflicting results. Akkasheh et al. reported the results of a pioneer clinical trial of probiotics used as a supplement in MDD patients [71]. In this double-blind, placebo-controlled study, MDD patients received placebos or supplement probiotic capsules containing *Lactobacillus acidophilus*, *Lactobacillus casei*, and *Bifidobacterium bifidum,* for eight weeks. At the end of the study period, patients receiving the probiotic capsules showed significant improvements in depression symptoms, as assessed by using the Beck Depression Inventory score [71]. More recently, a cohort study examined the effects of combined supplements of probiotics and magnesium orotate in a small group of selective serotonin reuptake inhibitor (SSRI)-resistant MDD patients [72]. At the end of the eight-week study period, the majority of the cohort demonstrated significantly improved depression symptoms and quality of life. However, relapse was observed while the patients were on a selective serotonin reuptake blocker but ceased to take the supplement probiotics and magnesium orotate [72]. However, a double-blind, randomized, placebo-controlled clinical trial that assessed the effects of probiotics as a primary treatment for MDD did not find that a combination of probiotics improved depression symptoms [73]. Participants of this trial were given placebo or probiotic formulas that contained *L. helveticus* R0052 and *B. longum* R0175 for eight weeks, and the severity of depression symptoms was assessed by the Montgomery-Åsberg Depression Rating Scale, the Improved Clinical Global Impressions Scale, and QIDS-SR16. Additionally, global function and abdominal symptoms were assessed, as well as the status of various biomarkers for inflammation. At the end of the eight-week study period, no significant difference was noted in any of the measurements in the psychological and functional outcomes or biomarkers between the placebo and probiotic groups. Overall, although treatment of MDD with probiotics showed some promise, because the number of clinical trials was very limited and a relatively small population was included in the available trials, it is difficult to conclude whether or not probiotics offer clinical benefits for MDD patients. More studies using larger populations will be required to fully examine the possibility of using probiotics as a treatment for MDD.

## CONCLUSIONS

Recent studies have recognized and underscored the importance of interactions between the brain and the gut, mediated by gut microbiota, in the pathogenesis of psychiatric disorders such as schizophrenia and MDD. Gut microbiota can affect the brain by releasing essential amino acids and participating in the immune and inflammatory responses. The brain, in turn, can modify the conditions of the microbial habitat and may influence the composition of the specific bacterial species present in the microbiota. Therefore, the microbiota has become a valid target in the search for effective treatments for schizophrenia and MDD. To date, only very few well-controlled clinical studies have been conducted, and the results from different clinical trials were not always consistent. Further studies are required to fully understand the intricate interactions between the members of the brain-gut axis and to validate/invalidate the clinical benefits of probiotics in the treatment of schizophrenia and MDD. Studies comparing the gut microbial composition of patients both during a psychiatric disorder episode (MDD or schizophrenia) and when in remission should shed light on the interactions between the gut flora and the brain during an active disease state. This type of study will also help to identify the species of bacteria that offer benefits to patients with psychiatric disorders. In order to understand the clinical benefits of probiotics, it will be helpful to establish to what extent the host gut flora could be modified by probiotic consumption, with or without dietary modification, and whether the effects are long-term. Furthermore, the molecular interactions between the brain and the gut flora could be explored if an established schizophrenia animal model with defined gastrointestinal microbial composition is available. Overall, more mechanistic studies are necessary for the development of probiotics as an effective therapy.
